# Identification of alkynyl nicotinamide HSN748 as a RET solvent-front mutant inhibitor with intracranial efficacy†

**DOI:** 10.1039/d5md00245a

**Published:** 2025-05-28

**Authors:** Ujjwol Khatri, Neetu Dayal, Kofi B. Owusu, Mandeep Kaur Hunjan, Shriya Pandey, Haley Anne Harper, Carli McMahan, Bennett D. Elzey, Tao Shen, Xueqing Hu, Kurt W. Evans, Ahmed El-Sheikh, Seong jun Jo, Frederick W. Holtsberg, M. Javad Aman, Funda Meric-Bernstam, Sukyung Woo, Herman O. Sintim, Jie Wu

**Affiliations:** a Department of Pathology, University of Oklahoma Health Sciences Oklahoma City OK 73104 USA jie-wu@ouhsc.edu; b Peggy and Charles Stephenson Cancer Center, University of Oklahoma Health Sciences Oklahoma City OK 73104 USA; c Department of Chemistry, Purdue University West Lafayette IN 47907 USA; d Department of Chemistry and Biochemistry, McCourtney Hall, University of Notre Dame 54417 Leahy Dr. IN 46556 USA hsintim@nd.edu; e Purdue University Institute for Cancer Research, Purdue University West Lafayette Indiana 47907 USA; f Department of Comparative Pathobiology, Purdue University West Lafayette Indiana 47907 USA; g Department of Investigational Cancer Therapeutics, The University of Texas MD Anderson Cancer Center Houston Texas 77030 USA; h Department of Pharmaceutical Sciences, School of Pharmacy & Pharmaceutical Sciences, State University of New York at Buffalo Buffalo NY 14214 USA; i KinaRx Inc, 4 Research Court Rockville MD 20850 USA; j Mike and Josie Harper Cancer Research Institute, 1234 N. Notre Dame Avenue South Bend IN 46617 USA

## Abstract

RET solvent-front G810C/R/S mutations confer resistance to the currently approved RET protein tyrosine kinase inhibitors (TKIs) selpercatinib and pralsetinib. Moreover, RET fusion-positive lung adenocarcinoma frequently metastasizes to the brain. To address these challenges, it is imperative to develop a RET TKI that is effective against solvent-front mutations and exhibits intracranial activity. We synthesized alkynyl nicotinamide-based RET TKIs and tested their efficacy in cell cultures in inhibiting selpercatinib/pralsetinib-resistant RET solvent-front mutants G810C/R/S found in cancer patients, and in BaF3/KIF5B-RET(G810C) cell-derived subcutaneous and intracranial tumors *in vivo*. We also evaluated alkynyl nicotinamide RET TKIs in KIF5B-RET-induced lung tumors in immune competent transgenic mice, and in CCDC6-RET fusion-positive thyroid patient-derived xenograft PDX.003.047 tumors. *In vivo* pharmacokinetics (PK) studies were conducted to determine drug concentrations in plasma and brain. HSN748, HSND19, and HSND14 demonstrated potent inhibition of RET G810C/R/S mutants, with low nanomolar IC_50_ values. HSN748 induced regression of subcutaneous B/KR(G810C) tumors without causing body weight loss. Both HSN748 and HSND19 significantly reduced KIF5B-RET-driven lung tumors in transgenic mice, and inhibited growth of CCDC6-RET-positive PDX tumors. Among three compounds (HSN748, HSND19, and HSN608) evaluated for B/KR(G810C) brain tumors, HSN748 exhibited significant intracranial tumor inhibition. PK analysis indicated that HSN748 has a brain/plasma partition coefficient (*K*_p_) of 0.4, demonstrating its capability to penetrate the central nervous system (CNS).

## Abstract 2

This study identified HSN748 as a potent RET kinase solvent-front mutant inhibitor with a high central nervous system (CNS) permeability.

## Graphical abstract

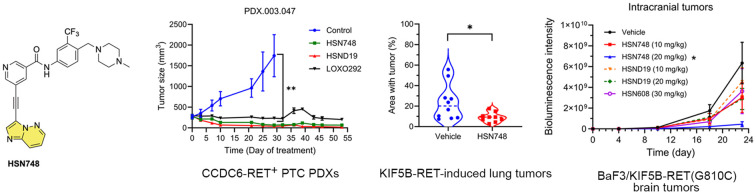

## 1. Introduction

Genetic alterations in the rearranged-during-transfection (RET) gene by chromosomal rearrangements or mutations result in constitutively active RET protein tyrosine kinase, which is a key target for cancer therapy.^1–3^ RET fusion oncogenes are mostly detected in non-small cell lung cancer (NSCLC) and papillary thyroid carcinoma (PTC). While various RET fusion partners have been identified in multiple cancer types, the KIF5B-RET fusion and the CCDC6-RET fusion are detected most frequently in NSCLC and PTC, respectively.^2^ Oncogenic RET mutations are found primarily in medullary thyroid cancer (MTC).^2^ In 2020, the US Food and Drug Administration (FDA) approved the RET protein tyrosine kinase inhibitors (TKIs) selpercatinib (LOXO292) and pralsetinib (BLU667) for advanced and metastatic RET-altered NSCLC and thyroid cancers. In 2022, selpercatinib received additional approval for RET fusion-positive solid tumors of any tissue origin after a tissue-agnostic trial.^4^

Despite these advances, an ongoing challenge in RET-targeted cancer therapy is the persistence of residual tumors in most patients, which often progresses to resistant disease over time.^2^ Both on-target and off-target mechanisms of resistance have been reported.^5–9^ The primary on-target resistance mechanism to both selpercatinib and pralsetinib involves RET solvent-front G810 to C, R, and S mutations,^6,8^ of which G810C is most frequently associated with tumor progression.^8^

The brain is a common metastasis site in NSCLC,^10^ and nearly half of RET fusion-positive NSCLC patients will experience central nervous system (CNS) metastases in their lifetime.^10–12^ This underscores the clinical need for CNS-penetrable compounds to develop the next generation of RET TKIs.

With RET solvent-front G810C/R/S mutations identified as the major on-target mechanism of acquired resistance to selpercatinib and pralsetinib, efforts are underway to develop RET solvent-front mutant effective TKIs.^2,13,14^ Previously, we characterized several alkynyl nicotinamide-based, type II RET TKIs for their efficacy in subcutaneous (s.c.) cell-derived xenograft (CDX) models of BaF3/KIF5B-RET(G810C) (B/KR(G810C) tumors.^13^ Here, we expanded the study to include HSN748, HSND19 and HSND14 in KIF5B-RET oncogene-induced lung tumors in transgenic mice and in B/KR(G810C) brain tumors. Our findings identify HSN748 as an orally available, CNS-penetrant RET solvent-front mutant inhibitor.

## 2. Results and discussion

### 2.1. Selpercatinib/pralsetinib-resistant RET solvent-front G810C/R/S mutants are sensitive to alkynyl nicotinamide-based compounds

**Fig. 1:**
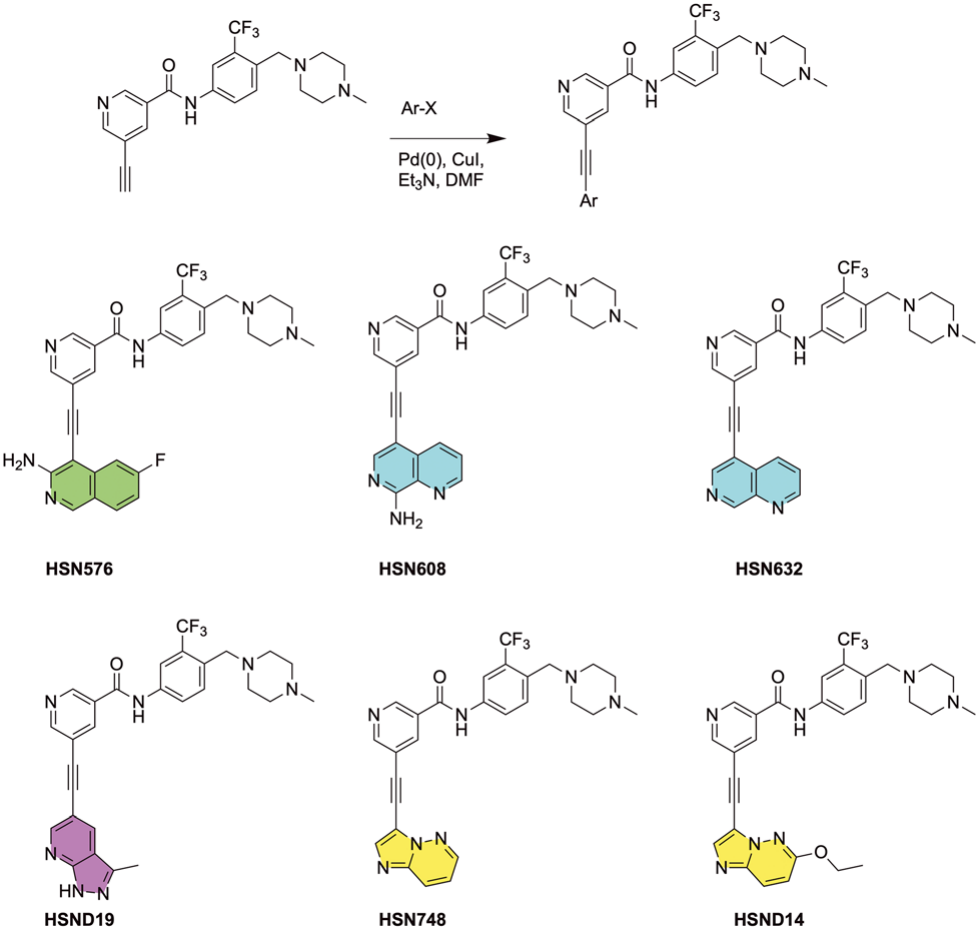
General synthetic scheme for the synthesis of compounds *via* Sonogashira reaction and chemical structures of compounds used in the study (HSN576, HSN608, HSN632, HSND19, HSN748, HSND14).

We reported previously that alkynyl nicotinamide-based RET kinase inhibitors HSN608, HSN632, and HSN576 were able to inhibit RET solvent-front G810C, G810R, and G810S mutants.^13^ We rationalized that keeping the alkynyl nicotinamide core but swapping the naphthyridine and isoquinoline hinge binding moiety in HSN608, HSN632 and HSN576 with other hinge binding moieties, such as imidazo[1,2-*b*]pyridazine or 1*H*-pyrazolo[3,4-*b*]pyridine could lead to other RET inhibitors with different physicochemical properties. Here, we reported three other alkynyl nicotinamide-based compounds HSN748 (containing imidazo[1,2-*b*]pyridazine hinge binder), HSND19 (containing 1*H*-pyrazolo[3,4-*b*]pyridine), and HSND14 (containing imidazo[1,2-*b*]pyridazine hinge binder) in BaF3/KIF5B-RET (B/KR) and mutant cell lines (Fig. 1). As shown in Table 1, HSN748, HSND19, and HSND14 inhibited the B/KR cells with low nanomolar IC_50_s that were slightly lower than that of selpercatinib, pralsetinib, zeteletinib, and vepafestinib. Importantly, while selpercatinib and pralsetinib IC_50_s for B/KR(G810C/R/S) cells ranged from 427 to 5150 nM, the HSN748, HSND19, and HSND14 IC_50_ values for these RET solvent-front mutants remained in the low nanomolar range (<33 nM) (Table 1). In comparison, the new RET kinase inhibitors zeteletinib and vepafestinib had G810C/R IC_50_s >100 nM. Immunoblotting analysis of cell lysates showed that HSN748, HSND19, and HSND14 were able to inhibit KIF5B-RET autophosphorylation and induce apoptosis at low nanomolar concentrations (Fig. S1A†). HSND19 and HSND14 also potently inhibited RET gatekeeper mutants V804M/L, whereas HSN748 had a modestly increased IC_50_s (88 nM and 55 nM) to these mutants (Table 1). Cabozantinib is a multiple kinase inhibitor used to treat RET-altered NSCLC patients prior to the development of selpercatinib and pralsetinib. Cabozantinib had modest potency on B/KR cells (IC_50_: 341 nM). Both RET solvent-front G810C/R/S and gatekeeper V804M/L mutants were resistant to cabozantinib (Table 1). Moreover, cabozantinib was 18-fold more potent in inhibiting TEL-VEGFR2 kinase-dependent cells than KIF5B-RET kinase-dependent cells, whereas selpercatinib and pralsetinib showed approximately 30-fold selectivity against VEGFR2. Zeteletinib and vepafestinib had the greatest selectivity against VEGFR2. HSN748, HSND19, and HSN14 had a similar potency between RET and VEGFR2 (Table 1, Fig. S1B†). Additional kinase sensitivity profiling has been reported.^15^ In non-cancerous IMR-90 human lung fibroblasts and the parental BaF3 cells, HSN748 displayed little inhibitory activity similar to selpercatinib, pralsetinib, and ponatinib (Fig. S4†).

**Table 1:** Sensitivity of test compounds on RET and RET solvent-front or gatekeeper mutants determined in B/KR and mutant cell lines

Compound	IC_50_ (nM)							VEGFR2/RET (fold)
	B/KR						B/TV	
	WT	G810C	G810R	G810S	V804M	V804L	WT	
HSN748	4.05 ± 0.18	7.93 ± 0.33	20.62 ± 1.42	13.88 ± 0.32	88.22 ± 3.34	55.73 ± 1.18	7.34 ± 0.09	1.81
HSND19	1.95 ± 0.12	9.08 ± 1.63	23.1 ± 1.39	4.00 ± 0.16	6.52 ± 0.45	20.6 ± 2.22	5.56 ± 1.11	2.85
HSND14	4.02 ± 0.19	15.93 ± 0.72	32.96 ± 0.67	5.21 ± 0.15	19.98 ± 0.89	29.99 ± 1.20	6.88 ± 0.15	1.71
Cabozantinib	341.2 ± 15.56	1533.78 ± 36.19	5150.80 ± 162.66	1096.77 ± 28.75	2453.77 ± 85.09	2143.67 ± 65.75	18.84 ±0.49	0.06
Selpercatinib	14.56 ± 0.48	1204.0 ± 123.7	1881.92.0 ± 96.03.0	611.34 ± 28.02	79.05 ± 4.83	30.51 ± 2.84	378.0 ± 14.78	26.03
Pralsetinib	21.06 ± 0.48	945.29 ± 45.19	2383.18 ± 208.21	427.10 ± 31.64	21.31 ± 0.81	34.95 ± 2.48	491.6 ± 14.38	23.34
Zeteletinib	16.68 ± 2.6	188.55 + 25.1	241.00 ± 28.4	73.09 ± 4.53	ND	ND	814.25 ± 194.75	49.04
Vepafestinib	19.41 ± 0.85	135.7 ± 83.16	164.65 ± 7.15	78.48 ± 1.51	ND	ND	1016 + 174	52.34

### 2.2. HSN748 causes regression of B/KR(G810C) CDX tumors at tolerable dose

**Fig. 2:**
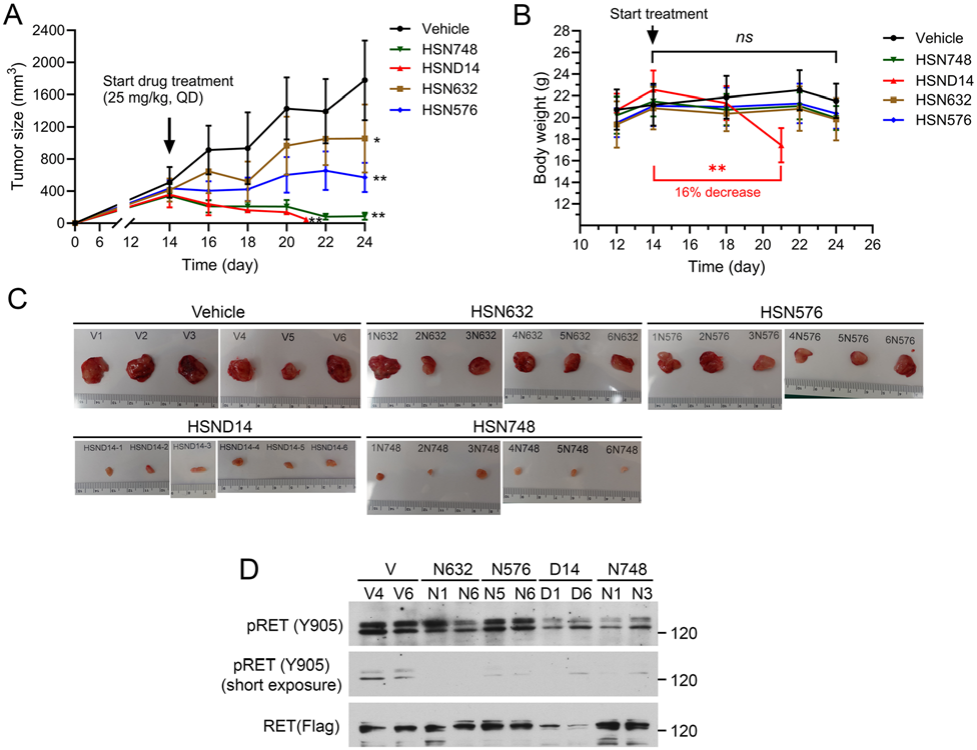
Anti-tumor activity of alkynyl nicotinamide compounds on subcutaneous B/KR(G180C) CDX. Mice with tumors (6 mice/group) were treated with indicated compound (25 mg kg^−1^, qd) or vehicle by oral gavage. The vehicle-treated mice were as reported^13^ and were performed in parallel with other treatments in the same experiment. (A) Tumor dimensions were measured with a caliper and the sizes were calculated as tumor size = (length × width × width)/2. The data are mean ± SD. (B) Mouse body weights were measured on the indicated days. (C) Images of dissected tumors collected at necropsy. (D) Immunoblotting of tumor tissue samples with the indicated antibodies.

We used B/KR(G810C) CDX tumors to test *in vivo* activity of alkynyl nicotinamide-based RET inhibitors because G810C is the most frequently detected secondary RET mutation in selpercatinib-treated patients who developed acquired resistance. After measurable s.c. B/KR(G810C) tumors were established, mice were treated (25 mg kg, qd) with HSN748, HSND14, HSN632, HSN576, or mock-treated with vehicle by oral gavage. *In vitro* properties of HSN632 and HSN576 have been described.^13^ Tumor size and animal body weight were monitored on the indicated dates as shown in Fig. 2A and B. HSN632 and HSN576 significantly reduced tumor growth, whereas HSN748 and HSND14 caused tumor regression (Fig. 2A). Weight loss was observed in HSND14 treated mice but not in other compound-treated mice. Consistently, examination of dissected tumors at necropsy and immunoblotting of tumor lysates showed that HSN748 and HSND14 were more effective in reducing tumor sizes and inhibiting RET phosphorylation (Fig. 2C and D). These results identified HSN748 as the most effective RET inhibitor for the B/KR(G810C) tumors at a tolerable dose in these animals.

### 2.3. HSN748 and HSND19 inhibit CCDC6-RET-positive thyroid PDX tumors

**Fig. 3:**
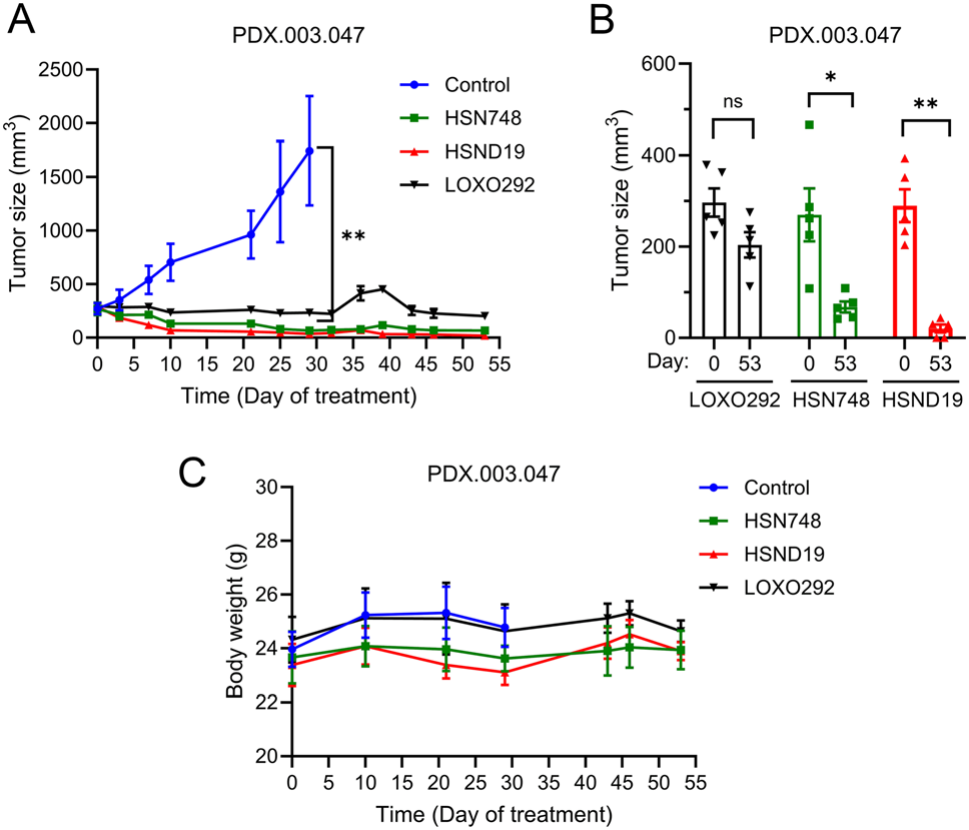
Inhibition of CCDC6-RET-positive thyroid PDXs by LOXO292, HSN748, and HSND19. PDX tumor fragments were implanted s.c. into nude mice and treated with indicated compounds once a day at 20 mg kg^−1^. (A) Tumor size measurements over the time course. The data are mean ± SEM. (B) Comparison of tumor sizes at the endpoint (day 53) with that at the baseline (day 0). (C) Mouse body weights on the indicated days.

We previously established a CCDC6-RET fusion-positive PDX, PDX.003.047, from a PTC patient. We next determined whether HSN748 and HSND19 could inhibit the growth of PDX.003.047 tumors. Female athymic nude mice bearing approximately 200 mm^3^ size of PDXs were either treated with vehicle or LOXO292, HSN748, or HSND19 (all 20 mg kg^−1^, qd, 5 mice/group). Tumor sizes and animal body weights were monitored as shown in Fig. 3, PDXs grew rapidly in the vehicle-treated group that reached the IACUC-allowed tumor size limit on day 29. LOXO292 suppressed the growth of PDX.003.047 tumors but did not significantly reduce the tumor size from the baseline, whereas HSN748 and HSND19 treatments significantly reduced the tumor sizes from their baselines that persisted to the endpoint on day 53 (Fig. 3B). All three compounds did not reduce animal body weights (Fig. 3C).

### 2.4. Anti-tumor activity of HSN748 and HSND19 in KIF5B-RET-driven lung tumors in transgenic mice

**Fig. 4:**
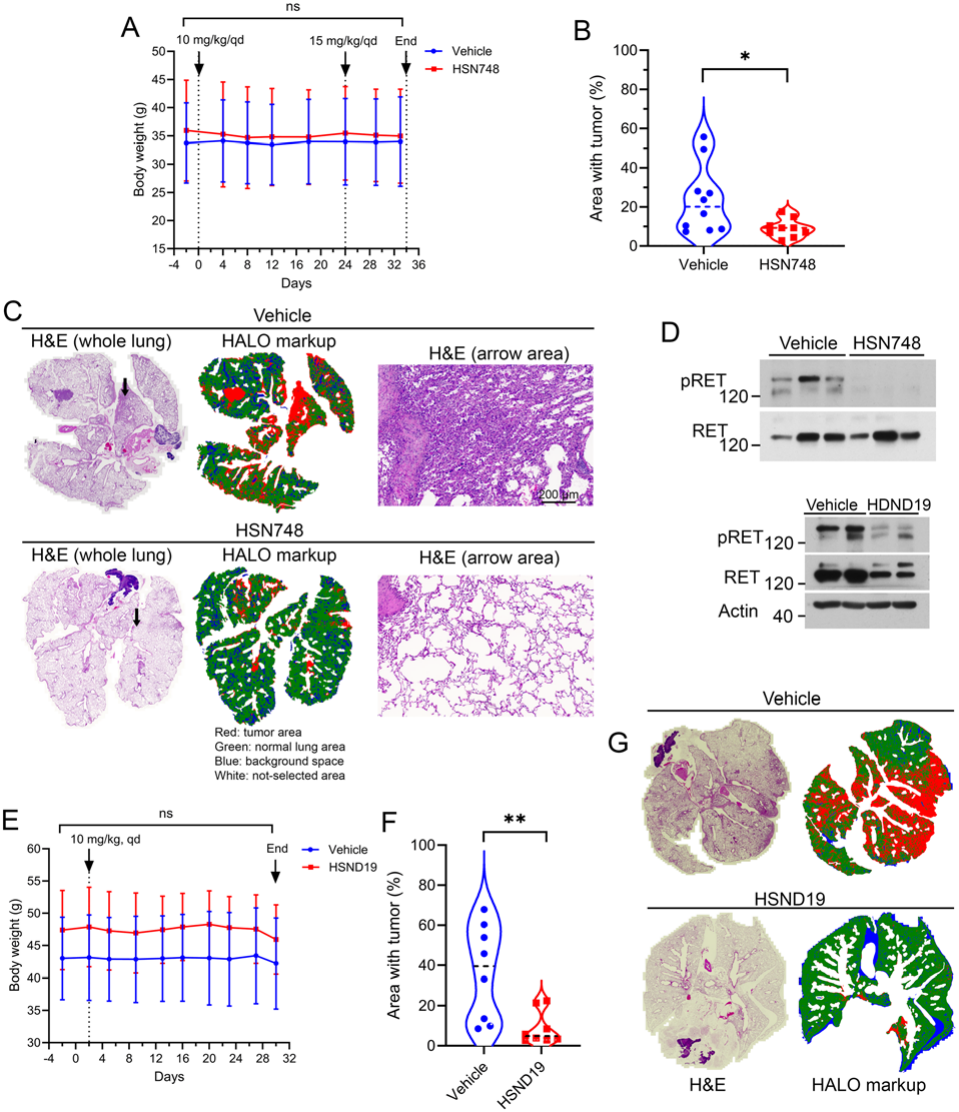
Inhibition of KIF5B-RET-driven lung tumors in transgenic mice by HSN748 or HSND19. (A–D) HSN748 treatment experiment. (E–G) HSND19 treatment experiment. (A and E) Dose schedule and body weight monitoring of HSN748 experiment. (B and F) Quantification of lung tissue areas with tumors. Each point represents a mouse. (C) Examples of H&E-stained lung tissue sections analyzed using HALO image analysis program for identification of tumor and non-tumor lung tissue areas. (D) Samples of lung tissue lysates were analyzed by immunoblotting with indicated antibody.

To determine whether alkynyl nicotinamide-based RET inhibitors can inhibit KIF5B-RET-induced lung tumors in immune-competent mice, we tested HSN748 and HSND19 in the C/KR transgenic mice.^16^ Expression of the KIF5B-RET oncogene was induced by feeding the C/KR bitransgenic animals with doxycycline (Dox), which led to the development of lung tumors. Animals were treated with HSN748, HSND19, or vehicle for 1 month as indicated in Fig. 4A and E in separate experiments (section 4.2.2 of Experimental). At the endpoint, lungs were collected as FFPE or as frozen tissues. H&E-stained lung tissue sections were quantitatively analyzed for areas of tumor lesion.

In the first experiment, the median lung tumor area in the vehicle-treated mice was 20.1%, which was significantly reduced to 9.2% in the HSN748-treated mice (Fig. 4B and C). Immunoblotting analysis of lung tissue samples showed that phospho-RET, which is indicative of active RET kinase activity, was present in the vehicle-treated lungs and was suppressed by HSN748 treatment (Fig. 4D). Body weight loss was not observed in these HSN748-treated transgenic mice (Fig. 4A). In the second experiment, HSND19 treatment reduced the median lung tumor burden from 39% to 4.84% in Dox-induced C/KR mice (Fig. 4F and G). Immunoblotting analysis showed that phospho-RET was reduced in the lungs of HSND19-treated mice (Fig. 4D).

### 2.5. Pharmacokinetics of HSND19 and HSN748

The *in vivo* PK properties of HSN748 and HSND19 were evaluated in rats following oral and/or IV administration. For HSND19, plasma concentration-time profiles were assessed after single IV and oral dosing (Fig. S2A†). After IV administration, the plasma concentration exhibited a biexponential decline. Following oral administration, the plasma concentration reached a peak concentration (*C*_max_) of 182 ng ml^−1^ at 4 hours and declined in parallel with the terminal phase of the IV profile, with an elimination half-life of 3.6 hours (Table 2). The oral bioavailability of HSND19 at 10 mg kg^−1^ was approximately 25%. HSND19 displayed a high volume of distribution (7.1 L kg^−1^), indicative of extensive tissue distribution. Brain distribution was evident, with a peak concentration of 25.5 ng ml^−1^ and total brain exposure (AUC_brain_) of 275 ng h ml^−1^ following a 2 mg kg^−1^ IV dose (Fig. S2B†).

**Table 2:** HSND19 pharmacokinetic parameters

PK parameter (unit)	Rat	
	IV	PO
	2 mg kg^−1^	10 mg kg^−1^
*t*_1/2_ (h)	3.6	3.7
*C*_max_ (ng ml^−1^)	308	182
*T*_max_ (h)	0.25	4
AUC_plasma_ (ng h ml^−1^)	1390	1758
*V*_ss_ (L kg^−1^)^a^	7.1	45.1
Cl (L h^−1^ kg^−1^)^a^	1.44	5.7
*F* (%)	—	25.3
AUC_Brain_ (ng h ml^−1^)	275	—

a *V*_ss_/*F* (L kg^−1^) or Cl/*F* (L h^−1^ kg^−1^) for oral doses.

For HSN748, oral PK was evaluated at three dose levels (20, 50, and 100 mg kg^−1^) in rats. Peak plasma concentrations were observed at 2–4 hours postdose, ranging from 911 ng ml^−1^ at 20 mg kg^−1^ to 3326 ng ml^−1^ at 100 mg kg^−1^ (Fig. S3A†). Exposure (*C*_max_ and AUC) increased slightly less than proportionally with dose escalation. Although the oral bioavailability of HSN748 was not directly determined in rats, dose-normalized AUC and *C*_max_ comparisons suggested it was higher than that of HSND19. A previous PK study in mice reported oral bioavailability of 60–80% at doses of 10–30 mg kg^−1^.^17^ The elimination half-life ranged from 3 to 6 hours (Table 3).

**Table 3:** HSN748 pharmacokinetic parameters

Parameter (unit)	Mouse	Rat		
	IV	PO		
	2 mg kg^−1^	20 mg kg^−1^	50 mg kg^−1^	100 mg kg^−1^
*t*_1/2_ (h)	1.3	3.05	4.26	5.84
*C*_max_ (ng ml^−1^)	513	911	1833	3326
*T*_max_ (h)	0.25	4	4	2
AUC_Plasma_ (ng h ml^−1^)	907	7525	17 786	33 549
AUC_Plasma_/dose ng h ml^−1^ per mg kg^−1^	435.5	376.3	355.7	335.5
*V*_ss_ (L kg^−1^)^a^	3.9	19.3	23.4	28.9
Cl (L h^−1^ kg^−1^)^a^	2.2	2.66	2.81	2.98
AUC_Brain_ (ng h ml^−1^)	377	—	—	—

a *V*_ss_/*F* (L kg^−1^) or Cl/*F* (L h^−1^ kg^−1^) for oral doses.

The brain distribution of HSN748 was assessed in mice following a 2 mg kg^−1^ IV dose (Fig. S3B†). HSN748 brain exposure (AUC_brain_ = 377 ng h ml^−1^) was approximately 42% of total plasma exposure (AUC_plasma_), with peak brain concentrations exceeding 198 ng ml^−1^. The compound exhibited a volume of distribution of 3.9 L kg^−1^, indicating efficient tissue penetration.

### 2.6. Intracranial efficacy of alkynyl nicotinamide-based RET inhibitors

**Fig. 5:**
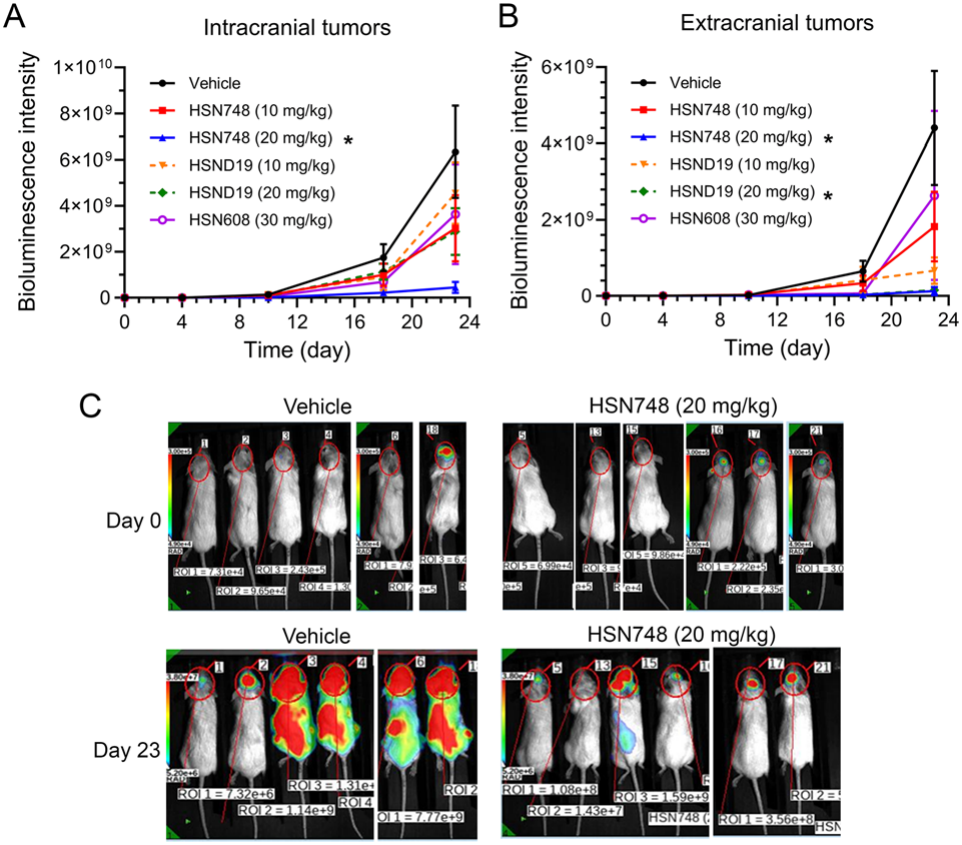
Intracranial efficacy of alkynyl nicotinamide-based RET TKIs. B/KR(G810C)-luc cells were injected into mouse brains and treated with indicated compounds. (A) Responses of intracranial tumors to the treatment were monitored by measurement of bioluminescence signal in the animal heads. The data are means and SEM. (B) Response of extracranial tumors were monitored by subtracting whole body bioluminescence signal with the bioluminescence signals of the head. (C) Bioluminescence images of vehicle- and 20 mg kg^−1^ HSN748-treated mice on day 0 and day 23 to illustrate the progression of tumors in vehicle-treated mice and suppression of tumor growth in HSN748-treated mic.

Approximately one-half of the RET fusion-positive NSCLC patients develop CNS metastases.^11^ Thus, CNS penetration is an important selection criterion in the development of new RET kinase inhibitors. To determine which of the alkynyl nicotinamide-based RET inhibitors have intracranial efficacy, we implanted B/KR(G180C)-luc cells into the brain of the mice, treated them with HSN748, HSND19, HSN608, or control vehicle and monitored the growth of B/KR(G810C) brain tumors by bioluminescence imaging (Fig. 5).

As reported by others, some of the intracranially injected tumor cells migrated to other parts of the body and grew as extracranial tumors, so that the bioluminescence signals in the animal head (intracranial) and the rest of the body (extracranial) were analyzed (Fig. 5). All drug treatments reduced the bioluminescence signal in the head. However, only HSN748 (20 mg kg^−1^) yielded statistically significant inhibition of the intracranial B/KR(G810C)-luc tumors (Fig. 5A and C). The extracranial bioluminescence signals of B/KR(G810C)-luc tumor cells were significantly inhibited by both HSND19 (20 mg kg^−1^) and HSN748 (20 mg kg^−1^) (Fig. 4B). Moreover, at the 10 mg kg^−1^ dose (Fig. 5A and B), HSN748 had a larger 52% inhibition of the intracranial tumors compared to a lower 28% inhibition by HSND19. In contrast, HSND19 gave a larger inhibition (85%) of the extracranial tumors than HSN748 did (58%). Although the differences at the 10 mg kg^−1^ dose level were not statistically significant, the trend of differences agreed with the notion that HSN748 has a higher intracranial efficacy.

*In vivo* PK studies showed that the brain-to-plasma partition coefficient (*K*_p_), calculated as AUC_brain_/AUC_Plasma_, was 0.42 for HSN748 and 0.2 for HSND19 (Tables 2 and 3). The higher *K*_p_ value for HSN748 indicates a greater proportion of the drug reaching the brain relative to plasma, consistent with its superior intracranial anti-tumor efficacy compared to HSND19. Additionally, with its higher oral bioavailability and total plasma exposure, HSN748 is expected to achieve greater CNS penetration.

CNS-penetrant, solvent-front G810 mutant-effective RET TKIs are needed to circumvent the acquired on-target mechanism of resistance to selpercatinib and pralsetinib. We have been developing alkynyl nicotinamide-based RET TKI for inhibiting RET solvent-front mutants. In a previous study, we reported that HSN608 and HSL468 were able to cause s.c. B/KR(G810C) tumor regression at a tolerable dose.^13^ Here, we expanded the study to new compounds. In addition to the s.c. CDX tumors, we evaluated the RET TKIs in CCDC6-RET-fusion-positive PDXs, in KIF5B-RET-driven lung tumors in transgenic mice, and in intracranial tumors.

In cell cultures, HSN748, HSND19, and HSND14 potently inhibited RET and RET solvent-front G810C, R, S mutants with low nanomolar IC_50_s similar to other compounds that we have reported.^13^ Among these three compounds, HSND19 appears slightly more potent than HSN748 and HSND14 on RET and slightly more selective against VEGFR2. In the s.c. B/KR(G810C) tumor experiment of HSN748, HSND14, HSN632 and HSN576, only HSN748 induced tumor regression without causing excessive animal body weight loss.

In the CCDC6-RET-positive thyroid PDX, LOXO292 (20 mg kg^−1^, qd) suppressed PDX growth while HSN748 and HSND19 at the same dose decreased the PDX tumor sizes from the baseline. In the KIF5B-RET-driven lung tumors of transgenic mice, both HSN748 and HSND19 significantly decreased lung tumor burden, indicating their effectiveness in inhibiting the RET fusion oncogene-driven lung tumors in their natural tumor microenvironment.

Both HSN748 and HSND19 demonstrated favourable *in vivo* PK properties, including oral bioavailability and elimination half-lives of 3–6 hours. Both compounds exhibited efficient tissue distribution, supported by predicted log *P* value of >1.5. HSN748 showed higher oral bioavailability and systemic exposure compared to HSND19.

Brain distribution of small compounds depends on factors like lipophilicity, molecular size, charge, blood–brain barrier (BBB) permeability, and efflux transporters (*e.g.*, P-glycoprotein), with metrics such as the brain-to-plasma partition coefficient (*K*_p_) and unbound drug fraction being critical. HSN748 demonstrated higher brain distribution (*K*_p_ = 0.42) compared to HSND19 (*K*_p_ = 0.2). A *K*_p_ of >0.3 is considered a high degree of CNS penetration. For comparison, the *K*_p_ of selpercatinib is 0.05 to 0.077 and the *K*_p_ of pralsetinib is 0.016–0.077.^18^

## 3. Conclusion

In this study, we have identified and demonstrated an alkynyl nicotinamide compound HSN748 as an orally available CNS-penetrant RET TKI with efficacy against selpercatinib/pralsetinib-resistant RET solvent-front mutants, showcasing promise for addressing CNS-involved RET-driven cancers. An advantage of HSN748 over HSND19 and the previously identified HSN608 was revealed in the B/KR(G810C)-luc brain tumor experiment. While HSND19 exhibited slightly lower IC_50_ in cell cultures and comparable efficacy in CCDC6-RET-dependent thyroid PDXs, KIF5B-RET-driven lung tumors in transgenic mice, and in B/KR(G810C)-luc extracranial tumors, only HSN748 displayed significant intracranial efficacy in B/KR(G810C)-luc brain tumors. In support of intracranial efficacy, PK studies revealed higher plasma and brain exposure for HSN748. Together, these results establish HSN748 as an orally bioavailable, CNS-penetrant RET TKI effective on RET solvent-front mutants.

## 4. Experimental

### 4.1. Chemistry

#### 4.1.1. General synthetic information

Unless specified otherwise, all reagents and solvents were sourced from commercial suppliers and used as received. Reactions were conducted in 20 mL screw-cap glass vials. ^1^H and ^13^C NMR spectra were recorded in chloroform-*d* or DMSO-*d*_6_ using a 500 MHz spectrometer, with tetramethylsilane as the internal standard. Purification was carried out using silica gel (230–400 mesh) *via* flash or column chromatography. Coupling constants (*J* values) are reported in Hertz (Hz). All compounds were characterized by ^1^H and ^13^C NMR, as well as high-resolution mass spectrometry (HRMS) data. HRMS were obtained through electrospray ionization (ESI) and analyzed using a TOF mass analyzer.

The synthesis and characterization of HSN608,^13^ HSN748,^17^ HSN632 (ref. 19) and HSN576 (ref. 20) have been previously reported.

#### 4.1.2. Sonogashira coupling general procedure for the synthesis of compound HSND14 and HSND19

In a 25 mL round-bottom flask, bromo substrate (1 mmol), alkyne substrate (1.1 mmol), PdCl_2_(PPh_3_)_2_ (3 mol%), XPhos (2 mol%), Cs_2_CO_3_ (3 equivalents), and CuI (1 mol%) were combined with anhydrous DMF (5 mL) and DIPEA (2.5 mL) under inert atmosphere. The reaction mixture was stirred at 60 °C overnight. Upon completion, the mixture was concentrated and extracted with ethyl acetate. The organic phase was washed with 2 N NaOH aqueous solution (20 mL) and brine (30 mL), then filtered through a Celite pad. The organic layer was dried over sodium sulfate, concentrated, and purified by silica gel column chromatography using DCM/MeOH (95 : 5) as the elution solvent to obtain the desired product.

#### 4.1.3. Synthesis of intermediate 3-bromo-6-ethoxyimidazo[1,2-*b*]pyridazine

3-Bromo-6-chloroimidazo[1,2-*b*]pyridazine (500 mg) was dissolved in ethanol (20 mL) and cooled to 0 °C. Sodium ethoxide (2.5 equivalents) was then added. The reaction mixture was stirred at room temperature overnight. Upon completion, the mixture was extracted with ethyl acetate and washed with brine. The organic phase was collected, dried over sodium sulfate, and concentrated under reduced pressure to yield the desired product. Off-white solid, 89% yield. ^1^H NMR (500 MHz, chloroform-*d*) *δ* 7.75 (dd, *J* = 9.6, 1.5 Hz, 1H), 7.58 (d, *J* = 1.4 Hz, 1H), 7.26 (s, 1H), 6.70 (dd, *J* = 9.6, 1.5 Hz, 1H), 4.47 (qd, *J* = 7.0, 1.4 Hz, 2H), 1.47 (td, *J* = 7.1, 1.5 Hz, 3H); ^13^C NMR (126 MHz, chloroform-*d*) *δ* 160.2, 137.8, 132.5, 127.3, 112.2, 100.7, 63.6, 14.2. HRMS (ESI) *m*/*z* calcd for C_8_H_9_BrN_3_O [M + H]^+^ 241.9929 found 241.9928.

#### 4.1.4. 5-((6-Ethoxyimidazo[1,2-*b*]pyridazin-3-yl)ethynyl)-*N*-(4-((4-methylpiperazin-1-yl)methyl)-3-(trifluoromethyl)phenyl)nicotinamide (HSND14)

Off-white solid, yield: 67%. ^1^H NMR (500 MHz, DMSO-*d*_6_) *δ* 10.89 (s, 1H), 9.12 (d, *J* = 2.1 Hz, 1H), 8.96 (d, *J* = 2.1 Hz, 1H), 8.52 (t, *J* = 2.2 Hz, 1H), 8.22 (d, *J* = 2.2 Hz, 1H), 8.16–8.03 (m, 3H), 7.71 (dd, *J* = 8.9, 3.2 Hz, 1H), 7.02 (d, *J* = 9.6 Hz, 1H), 4.45 (q, *J* = 7.0 Hz, 2H), 3.62 (d, *J* = 4.3 Hz, 2H), 2.88 (s, 4H), 2.62–2.51 (m, 7H), 1.40 (td, *J* = 7.0, 2.5 Hz, 3H); ^13^C NMR (126 MHz, DMSO-*d*_6_) *δ* 163.8, 160.6, 154.0, 148.8, 139.9, 138.5, 137.9, 137.5, 132.3, 131.9, 130.3, 128.5, 127.9 (q, *J* = 30.2 Hz), 125.7 (q, *J* = 273.4 Hz), 124.0, 119.2, 117.8, 114.3, 111.8, 94.8, 81.3, 63.8, 57.3, 53.7, 50.8, 43.7, 14.6; HRMS (ESI) *m*/*z* calcd for C_29_H_29_F_3_N_7_O_2_ [M + H]^+^ 564.2335 found 564.2339.

#### 4.1.5. 5-((3-Methyl-1*H*-pyrazolo[3,4-*b*]pyridin-5-yl)ethynyl)-*N*-(4-((4-methylpiperazin-1-yl)methyl)-3-(trifluoromethyl)phenyl)nicotinamide (HSND-19)

Off-white solid (69 mg, 52%); ^1^H NMR (500 MHz, DMSO-*d*_6_) *δ* 10.85 (s, 1H), 9.10 (d, *J* = 2.2 Hz, 1H), 8.96 (d, *J* = 2.0 Hz, 1H), 8.69 (d, *J* = 1.9 Hz, 1H), 8.53 (dt, *J* = 4.8, 2.3 Hz, 2H), 8.22 (d, *J* = 2.4 Hz, 1H), 8.09 (dd, *J* = 8.7, 2.3 Hz, 1H), 7.72 (d, *J* = 8.5 Hz, 1H), 3.66 (s, 2H), 3.29–2.79 (m, 8H), 2.71 (s, 3H), 2.52 (s, 3H); ^13^C NMR (126 MHz, DMSO-*d*_6_) *δ* 163.9, 154.3, 151.7, 151.4, 148.5, 142.3, 138.6, 137.8, 133.5, 133.5, 132.0, 130.2, 127.9 (q, *J* = 28.9 Hz), 125.7 (q, *J* = 273.4), 124.0, 119.6, 117.8, 114.0, 110.5, 92.0, 86.6, 57.0, 53.2, 49.9, 12.6; HRMS (ESI) *m*/*z* calcd for C_28_H_27_F_3_N_7_O [M + H]^+^ 534.223, found 534.2224.

### 4.2. Biological evaluation

#### 4.2.1. Cell lines and cell viability assay

BaF3 cell lines expressing a flag-tagged KIF5B-RET (B/KR) and the KIF5B-RET containing G810C, G810R, G810S, V804M, V804L mutations were as reported.^8,13,17,21–23^ To generate B/KR(G810C) cells expressing dual-luciferase/GFP genes [B/KR(G810C)-Luc], lentivirus was prepared from pCDH-EF1a-eFFly-eGFP plasmid (Addgene # 104834)^24^ and used to infect B/KR(G810C) cells as described.^16^ Infected cells were cultured for two weeks, and cells with high GFP intensity were sorted into a 96-well plate (1 cell/well). After the expansion of growing cell lines, they were screened for a high level of luciferase activity by luminescence imaging after exposure to D-luciferin. IMR-90 cells were from ATCC.

To generate BaF3 cells expression a flag-tagged TEL-VEGFR2, a cDNA encoding TEL-VEGFR2, which produces a protein of TEL (ETV6) amino acids 1–336 fused to VEGFR2 (KDR) amino acids 807–1356, was synthesized chemically (Genescript). The TEL-VEGFR2 cDNA was then subcloned into pLentP lentiviral vector.^21^ and BaF3/TEL-VEGFR2 (B/TV) cells were generated by infecting BaF3 cells with the lentiviral TEL-VEGFR2 as described. Cell viability assay was performed using CellTiter-Glo reagent (G7573, Promega, Madison, WI, USA) after 3 days of drug treatment as described for BaF3-derived cells and 5 days for IMR-90 cells.

#### 4.2.2. Tumor inhibition studies in animals

All animal procedures were performed in accordance with the Guidelines for Care and Use of Laboratory Animals of University of Oklahoma Health Sciences, Purdue University, or the MD Anderson Cancer Center and approved by the Animal Ethics Committee of University of Oklahoma Health Sciences, Purdue University, or the MD Anderson Cancer Center. B/KR(G810C) cells were tested free of mycoplasma and mouse pathogens. Subcutaneous B/KR(G810C) CDX experiment was performed in parallel with other compounds reported previously.^13^ Briefly, B/KR(G810C) cells (1 × 10^7^ cells/0.1 ml per each) were inoculated s.c. into the right flank of 5-week-old female SHO mice (Charles Rivers, Wilmington, MA, USA). After measurable tumors were formed, mice were treated with test compounds or vehicle by oral gavage as specified in Fig. 2. Tumor size and animal body weight were measured with a caliper and a scale as described.^8,13^

The patient provided informed written consent for sampling for PDX generation by image-guided biopsy. This study was approved by the Institutional Review Board at the University of Texas MD Anderson Cancer Center (PA13-0592). PDX establishment methodology was previously described.^25,26^ Tumor fragments were implanted into 5 to 7 weeks old female athymic nude mice. Briefly, a 0.3 cm incision was made, and an approximately 27 mm^3^ tumor fragment was inserted into the subcutaneous pocket. The skin was then closed with wound clips. Mice were randomized and treated when the tumor volume reached at least 200 mm^3^. Tested drugs were dissolved in DMSO and then suspended as 5% DMSO : 40% PEG300 : 5% TWEEN80 : 50% water. For all drug, 100 μl was given to each mouse by oral gavage. Tumor volume was calculated as described.^13^

For the HSN748 treatment experiment in CCSP-rtTA/tetO-KIF5B-RET(C/KR) transgenic mice,^16^ approximately 2 months old male and female C/KR transgenic mice were fed with rodent chow containing 200 mg kg^−1^ doxycycline (Dox Diet, Bio-Serv) for 4 months to induce KIF5B-RET-driven lung tumors. The doxycycline-induced transgenic mice were then treated with HSN748 or vehicle by oral gavage as indicated in Fig. 3A. For the HSND19 treatment experiment in C/KR transgenic mice, 3 months old male and female C/KR transgenic mice were induced with doxycycline for 6 months as above and then treated with HSND19 or vehicle by oral gavage as indicated in Fig. 3E. At the endpoint, mouse lungs were collected and either fixed in 10% buffered formalin or snap-frozen in liquid nitrogen as described.^16,27^

For histological assessment of lung tumor burden and response to treatment, sections (4 μm thick) of formalin-fixed paraffin-embedded lung tissues were stained with hematoxylin and eosin (H&E). Two H&E-stained tissue sections that were 5 slides apart were scanned using an Axioscan Zeiss slide scanner at 20X magnification. The scanned lung images were then analyzed with the HALO® image analysis program (v3.6, Indica Labs). The random forest machine learning algorithm of HALO® was used to identify tumor *vs.* normal area by using the tissue classifier add-on. Tumor and normal areas were classified using normal mouse lungs as the reference. Fibrotic stroma was excluded.

Intracranial tumor experiments were performed using female NRG mice (Jackson Laboratory stock #007799) 10–12 weeks old as the hosts. Mice were anesthetized with 3% isoflurane gas and situated in a stereotactic frame (Stoelting). To determine the injection site, the scalp was opened with a scalpel and the bregma was located. Then the point 1.5 mm posterior and 2 mm to the right was identified and a 2 mm burr hole was drilled just through the skull. 3 × 10^5^ B/KR(G810C)-Luc cells in 3 μL were injected into the brain at a depth of 4 mm over 3 minutes using a 10 μL Hamilton syringe. The syringe was withdrawn over 5 minutes and the hole was filled with bone wax, the scalp was closed with medical glue, and the mice were returned to a heated cage for recovery. Tumor growth was determined *via* bioluminescent imaging with a Spectral Ami Optical Imaging System (Bruker). Mice were anesthetized with 3% isoflurane gas and injected intraperitoneally with 150 mg kg^−1^d-luciferin. After 15 minutes, mice were imaged for photon emission to determine tumor burden.

#### 4.2.3. *In vivo* pharmacokinetic analysis

For compound HSND19, male SD rats received an intravenous (IV) dose of 2 mg kg^−1^ and an oral (PO) dose of 10 mg kg^−1^. Both formulations were prepared in 10% DMSO/90% (20% HP-β-CD in water, w/w). Blood samples were collected at 0.25, 0.5, 1, 2, 4, 8, and 24 hours post-dosing. For compound HSN748, male SD rats received oral doses of 20, 50, and 100 mg kg^−1^ in a solution of 0.5% methylcellulose (4000 cp)/0.1 M citrate buffer (pH 4.5). Blood samples were collected at 0.25, 0.5, 1, 2, 4, 6, 8, and 24 hours post-dosing. For IV administration, male CD1 mice received 2 mg kg^−1^ prepared with 5% DMSO/5% Tween80/40% PEG400/50% PBS, with sampling at 0.083, 0.25, 0.5, 1, 2, 4, 8, and 24 hours.

In oral PK studies, blood samples were collected at each time point, with a total of 3 animals per study. In IV studies, animals were euthanized after blood collection at each time point to collect brain tissue, using a total of 21 animals per study. Whole blood samples were collected in heparinized tubes and centrifuged to separate plasma. Brain samples were rinsed, weighed, and immediately frozen in liquid nitrogen, and stored at −80 °C until analysis. The PK studies were conducted by Pharmaron (Beijing, China). Animal procedures were performed in accordance with the Guidelines for Care and Use of Laboratory Animals of Pharmaron (Beijing, China) and approved by the Animal Ethics Committee of Pharmaron (Beijing, China).

The mean concentration-time profiles were analyzed using Phoenix WinNonlin (version 8.4; Certara USA, Inc., Princeton, NJ) to calculate standard PK parameters, including the area under the curve from zero to infinity (AUC), bioavailability (*F*), clearance, volume of distribution, and half-life (*t*_1/2_).

#### 4.2.4. Statistical analysis

Statistical analysis was performed using the student's unpaired *t*-test with Welch's correction. *p* < 0.05 was considered statistically significant. The data and statistical analysis comply with the recommendations on experimental design and analysis in pharmacology.^28^ Animal body weight changes were analyzed using one-way ANOVA or Kruskal–Wallis based on normality test.

## Author contributions

UK: data curation, formal analysis, validation, investigation, writing – original draft, writing – review and editing. ND: data curation, validation, investigation, writing – review and editing. KBO: data curation, formal analysis, validation, investigation, writing – review and editing. MKH: data curation, validation, investigation, writing – review and editing. SP: data curation, investigation, writing – review and editing. HAH: data curation, formal analysis, investigation, writing – review and editing. CM: data curation, investigation, writing – review and editing. BDE: data curation, supervision, writing – review and editing. TS: data curation, investigation, writing – review and editing. XH: data curation, investigation, writing – review and editing. KWE: data curation, investigation, writing – review and editing. AE-S: data curation, investigation, writing – review and editing. SjJ: data curation, investigation, writing – review and editing. FWH: funding acquisition, project administration, writing – review and editing. MJA: funding acquisition, project administration, writing – review and editing. FM: funding acquisition, project administration, writing – review and editing. SW: funding acquisition, project administration, writing – review and editing. HOS: conceptualization, resources, data curation, formal analysis, supervision, funding acquisition, validation, investigation, visualization, methodology, writing – original draft, project administration, writing – review and editing. JW: conceptualization, resources, data curation, formal analysis, supervision, funding acquisition, validation, investigation, visualization, methodology, writing – original draft, project administration, writing – review and editing.

## Conflicts of interest

HOS, MJA, and FWH hold shares in KinaRx Inc, a company developing potential cancer therapeutics. Other authors declare no conflict of interest.

## Supplementary Material

MD-016-D5MD00245A-s001

## Data Availability

All data are contained in the manuscript and ESI.†
