# Supplementary material for: Identification of alkynyl nicotinamide HSN748 as a RET solvent-front mutant inhibitor with intracranial efficacy
Source: RSC Med Chem. 2025 May 28;16(8):3541–50. doi: 10.1039/d5md00245a (PMC12147034; doi:10.1039/d5md00245a)
Supplement: MD-016-D5MD00245A-s001 [file MD-016-D5MD00245A-s001.pdf]

Supplementary Information (SI) for RSC Medicinal Chemistry.
This journal is © The Royal Society of Chemistry 2025

Supplementary Information

Identification of alkynyl nicotinamide HSN748 as a RET solvent-front mutant inhibitor with intracranial efficacy

Ujjwol Khatri[1,2], Neetu Dayal[3], Kofi B. Owusu[4], Mandeep Kaur Hunjan[3], Shriya Pandey[1,2], Haley Anne Harper[5], Carli McMahan[5], Bennett D. Elzey[5,6], Tao Shen[1,2], Xueqing Hu[1,2], Kurt W. Evans[7], Ahmed El-Sheikh[8], Seong jun Jo[8], Frederick W. Holtsberg[9], M. Javad Aman[9], Funda Meric-Bernstam[7], Sukyung Woo[8], Herman O. Sintim[3,4,9,10*], and Jie Wu[1,2,*]

[1]Department of Pathology, University of Oklahoma Health Sciences, Oklahoma City, OK 73104, USA

[2]Peggy and Charles Stephenson Cancer Center, University of Oklahoma Health Sciences, Oklahoma City, OK 73104, USA

[3]Department of Chemistry, Purdue University, West Lafayette, IN 47907, USA

[4]Department of Chemistry and Biochemistry, McCourtney Hall, 54417 Leahy Dr., University of Notre Dame, IN 46556, USA

[5]Purdue University Institute for Cancer Research, Purdue University, West Lafayette, Indiana 47907, USA

[6]Department of Comparative Pathobiology, Purdue University, West Lafayette, Indiana 47907, USA

[7]Department of Investigational Cancer Therapeutics, The University of Texas MD Anderson Cancer Center. Houston, Texas, 77030, USA

[8]Department of Pharmaceutical Sciences, School of Pharmacy & Pharmaceutical Sciences, State University of New York at Buffalo, Buffalo, NY 14214, USA

[9]KinaRx Inc, 4 Research Court, Rockville, MD 20850, USA

[10]Mike and Josie Harper Cancer Research Institute, 1234 N. Notre Dame Avenue, South Bend, IN 46617 USA

*Correspondence:

Herman O. Sintim, Department of Chemistry and Biochemistry, University of Notre Dame, McCourtney Hall, Room 305A, 54417 Leahy Dr., Notre Dame, IN 46556, USA, Tel. 574-631-2014 email: hsintim@nd.edu, or Jie Wu, University of Oklahoma Health Sciences, 975 NE 10th Street, BRC413, Oklahoma City, OK 73104, USA, Tel. 405-271-8001 ext. 31092, email: jie-wu@ouhsc.edu

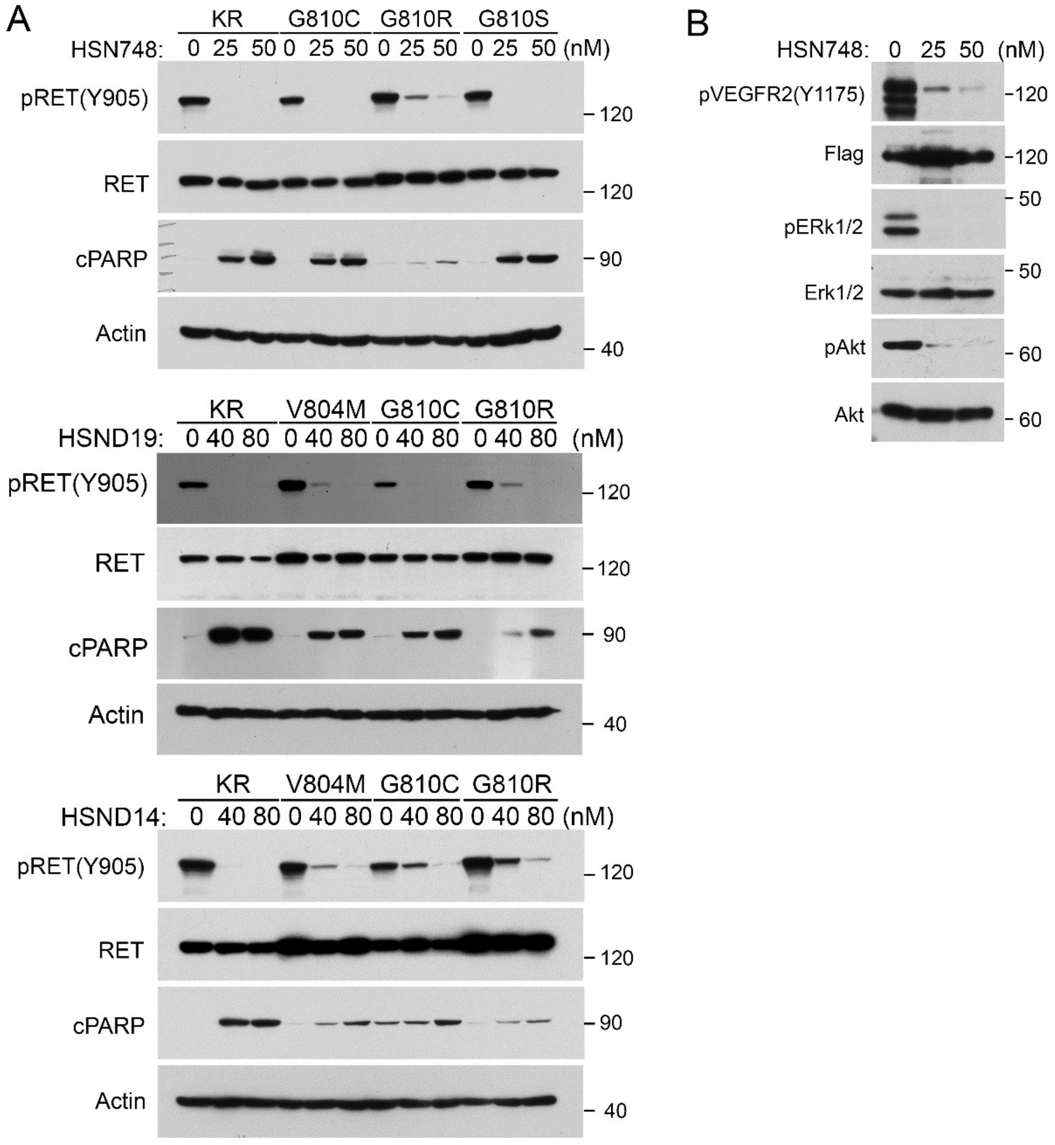

Figure S1. Immunoblotting analyses of cell lysates.
(**A**) Immunoblotting analysis of compounds on inhibition of KR or its mutant kinase activity and induction of apoptosis in B/KR and mutant cells. (**B**) Immunoblotting analysis of HSN748-treated B/TV cells.

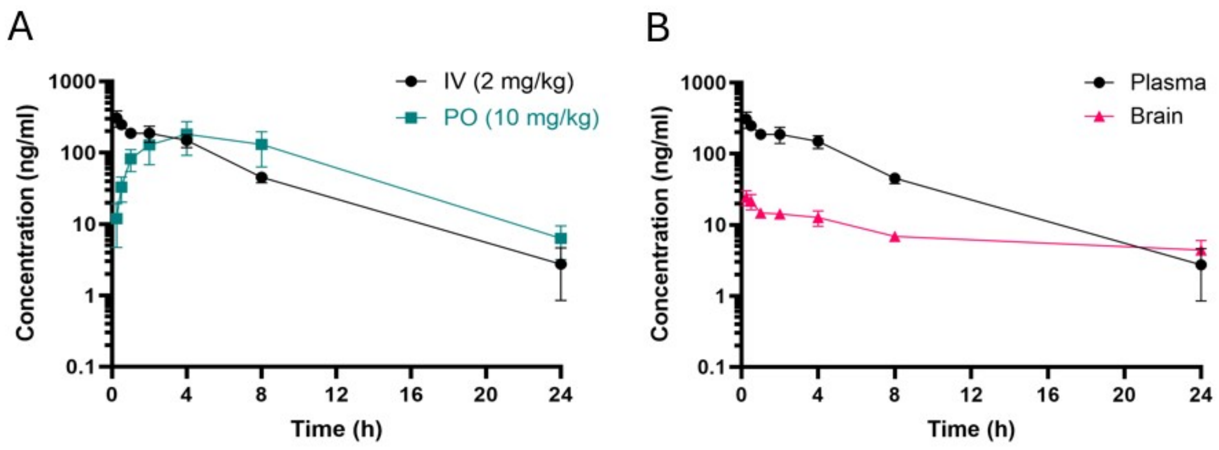

Figure S2. Pharmacokinetics of HSND19 in rats.
(**A**) Plasma concentration-time profiles of HSND19 in rats following an intravenous (IV) dose of 2 mg/kg and an oral (PO) dose of 10 mg/kg. (**B**) Concentration-time profiles of HSND19 in plasma and brain following an IV dose of 2 mg/kg in rats. Each data point represents the mean ± SD from three rats, except for the last time point in the brain profile, where one concentration was below the limit of quantification. The IV plasma profile in panel A is derived from the IV PK study shown in panel B.

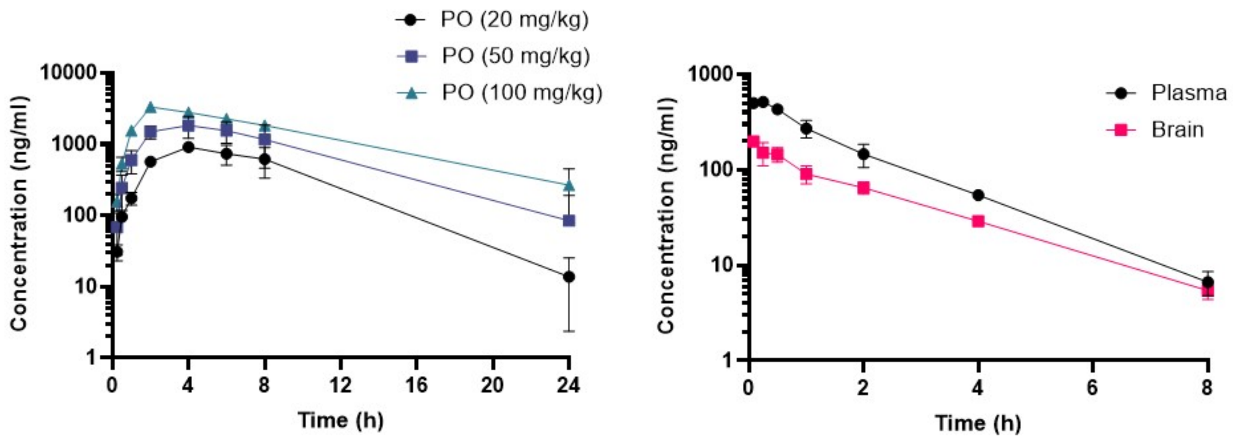

Figure S3. Pharmacokinetics of HSN748 in rats and mice.
(**A**) Plasma concentration-time profiles of HSN748 in rats following oral doses of 20, 50, and 100 mg/kg. (**B**) Concentration-time profiles of HSN748 in plasma and brain following an IV dose of 2 mg/kg in mice. Each data point represents the mean ± SD from three animals.

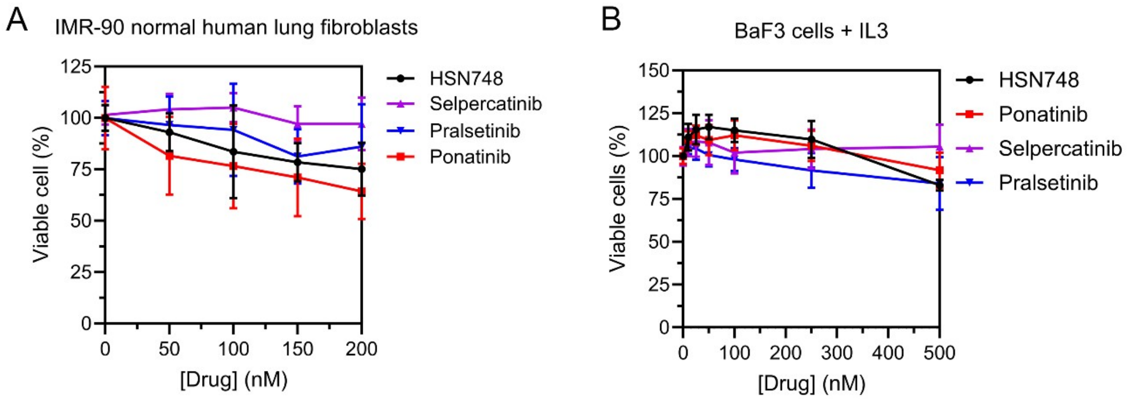

Figure S4. Activity of HSN748 in normal human fibroblasts and IL3-dependent BaF3 cells
(**A**) IMR-90 normal human lung fibroblast cells were plated in 96-well plates and treated with indicated drugs for 5 days. (**B**) The non-transformed, IL-3-dependent BaF3 cells were cultured in medium with 0.5 nM mouse IL-3 in 96-well plates and treated as in (**A**) for 3 days. Viable cells were then determined using CellTiter-glo reagent. Data were from two triplicate experiments.

^{1}H and ^{13}C spectra's

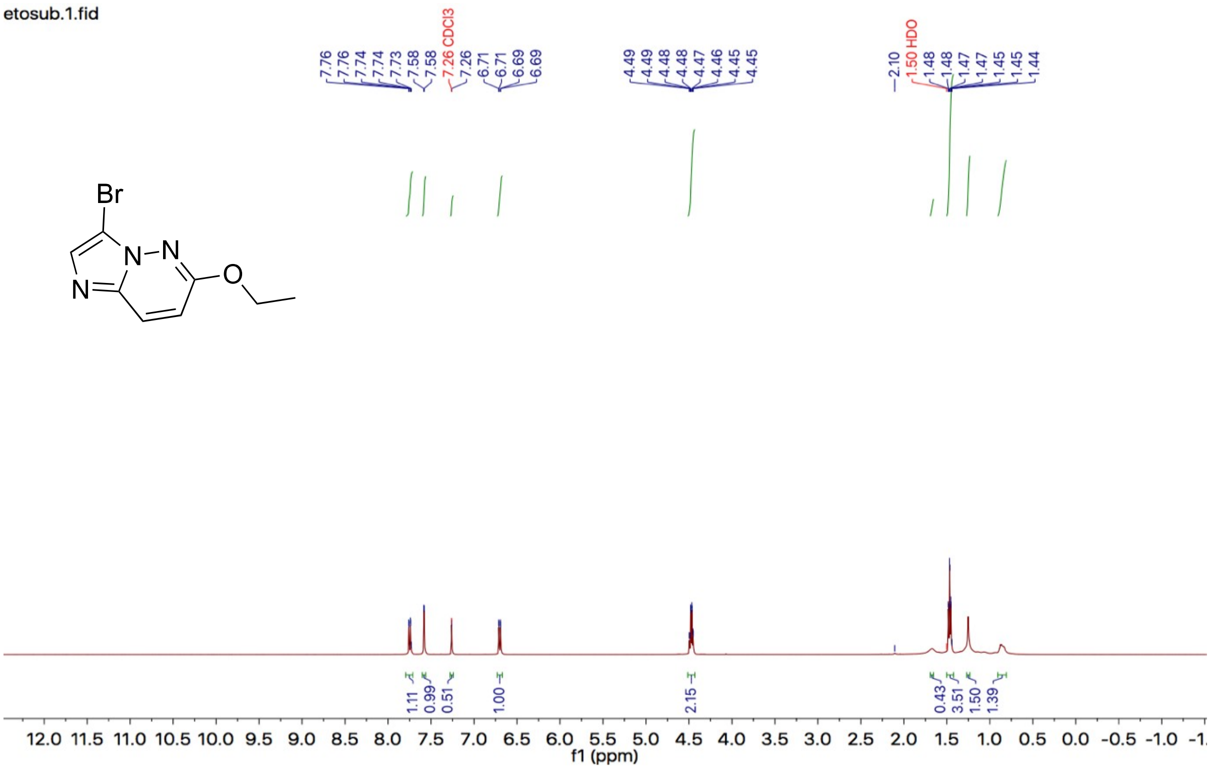

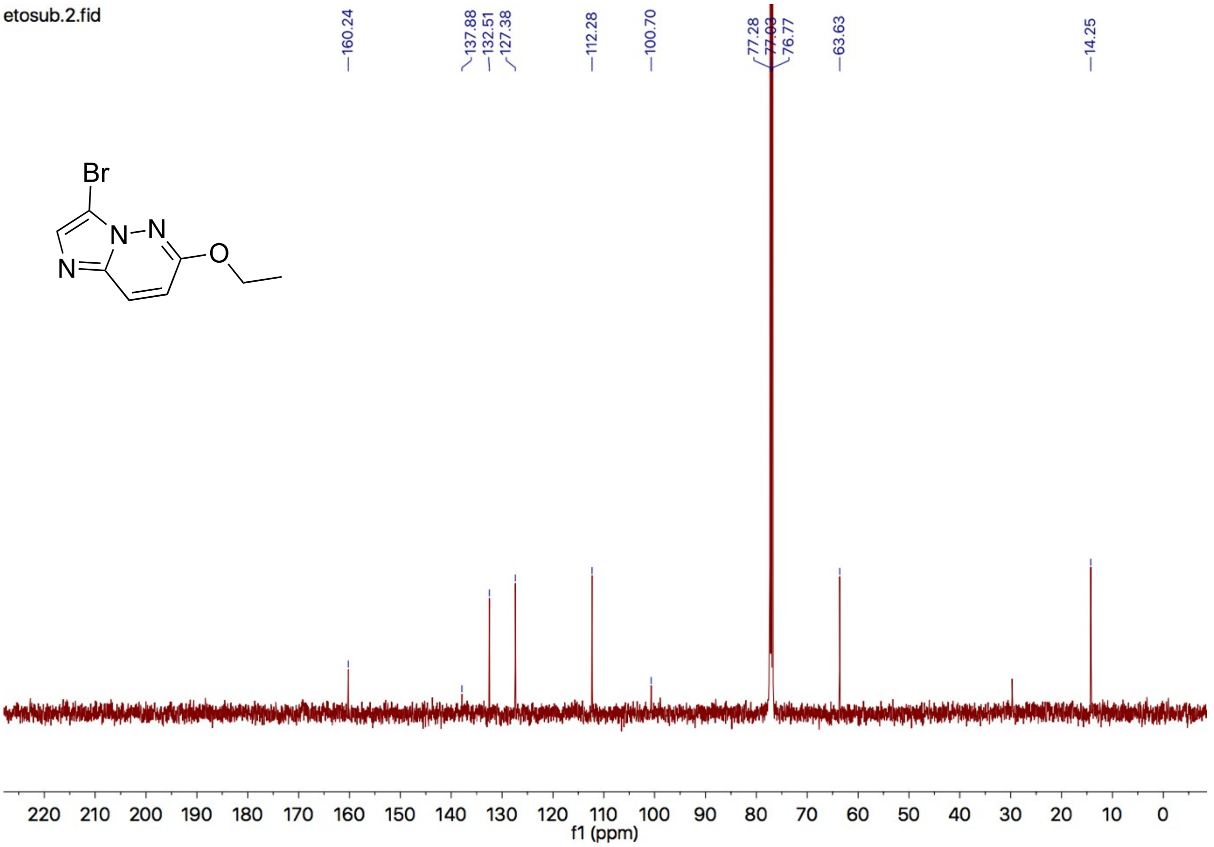
etosub.2.fid
160.24
137.88
132.51
127.38
112.28
100.70
77.28
77.03
76.77
63.63
14.25
Br
N
N
N
O
220 210 200 190 180 170 160 150 140 130 120 110 100 90 80 70 60 50 40 30 20 10 0
f1 (ppm)

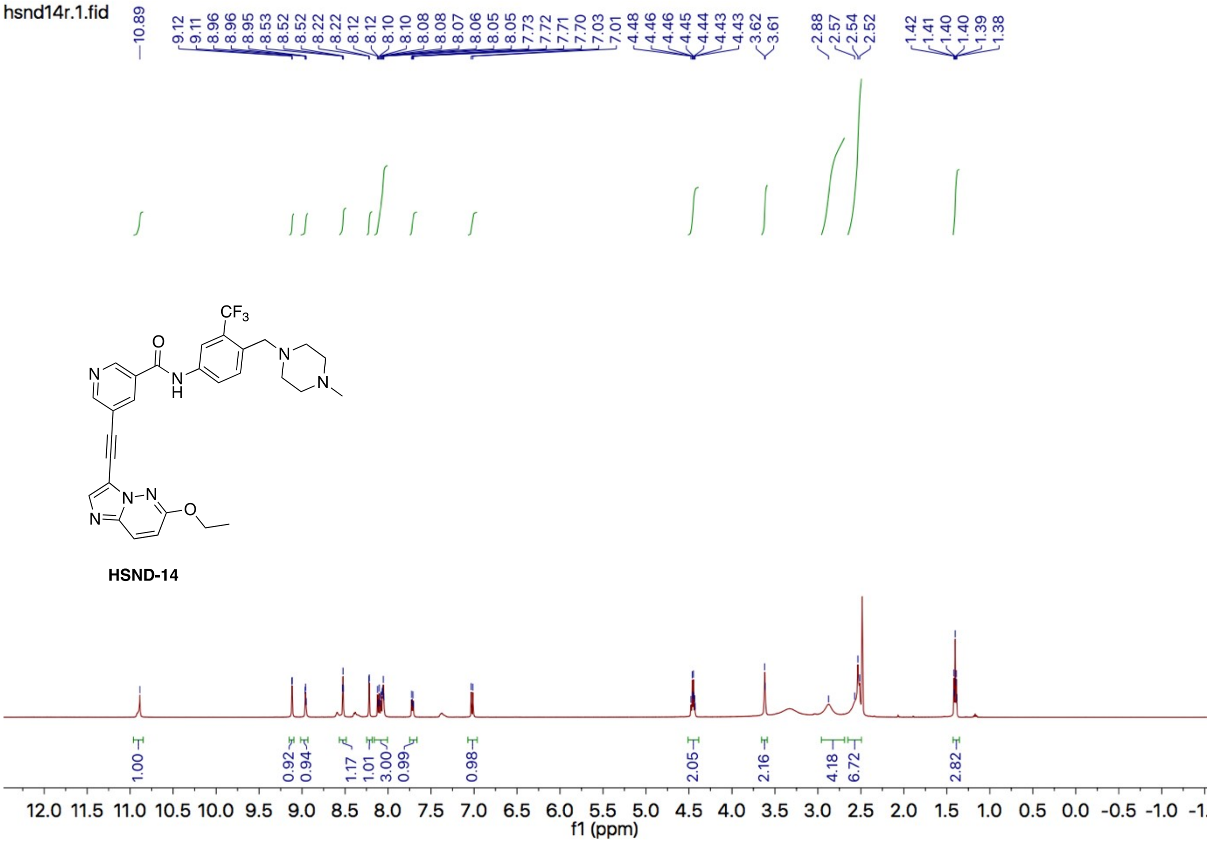
hsnd14r.1.fid
10.89
9.12 9.11 8.96 8.95 8.53 8.52 8.52 8.22 8.22 8.12 8.12 8.10 8.10 8.08 8.07 8.06 8.05 8.05 7.73 7.72 7.71 7.70 7.03 7.01
4.48 4.46 4.45 4.44 4.43 4.43
3.62 3.61
2.88 2.57 2.54 2.52
1.42 1.41 1.40 1.40 1.39 1.38
CF3
O
N
N
H
N
N
N
N
N
O
HSND-14
1.00
0.92
0.94
1.17
1.01
3.00
0.99
0.98
2.05
2.16
4.18
6.72
2.82
12.0 11.5 11.0 10.5 10.0 9.5 9.0 8.5 8.0 7.5 7.0 6.5 6.0 5.5 5.0 4.5 4.0 3.5 3.0 2.5 2.0 1.5 1.0 0.5 0.0 -0.5 -1.0 -1.
f1 (ppm)

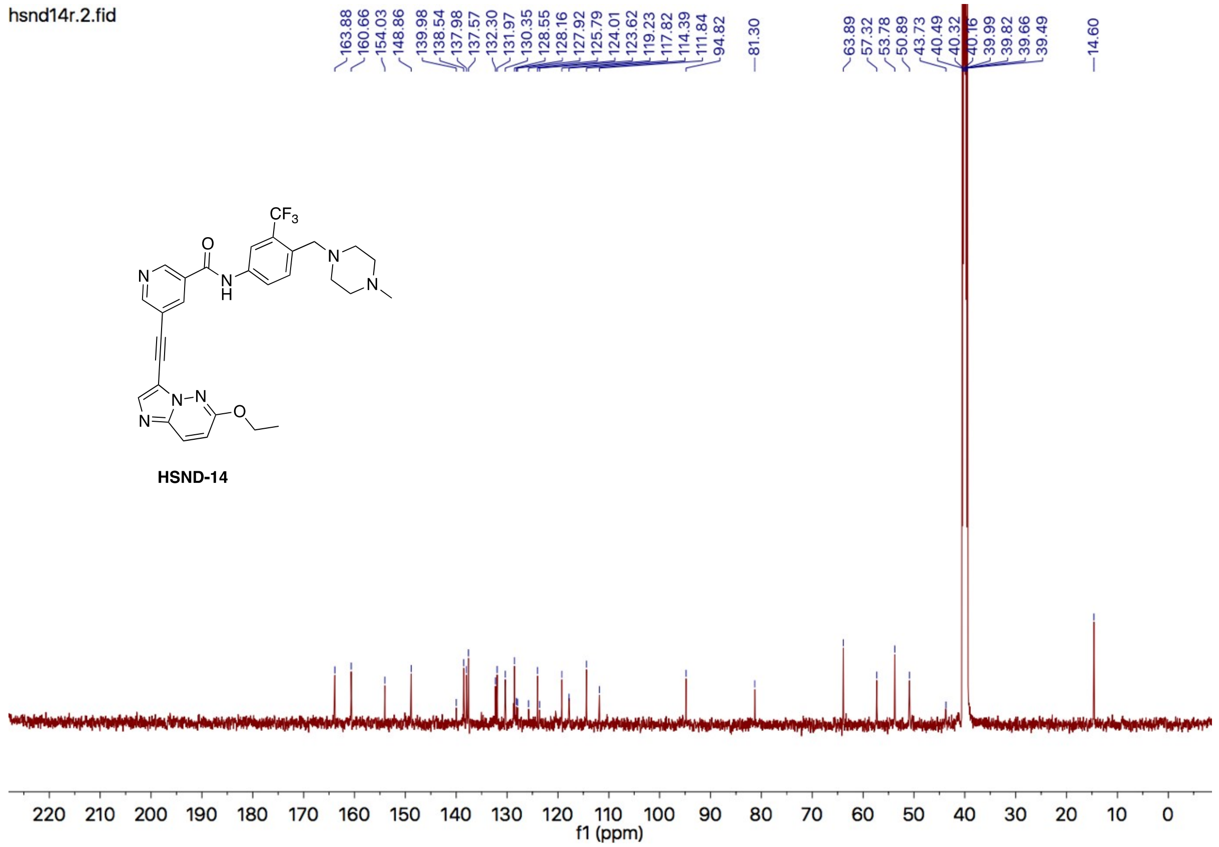

HSND-14

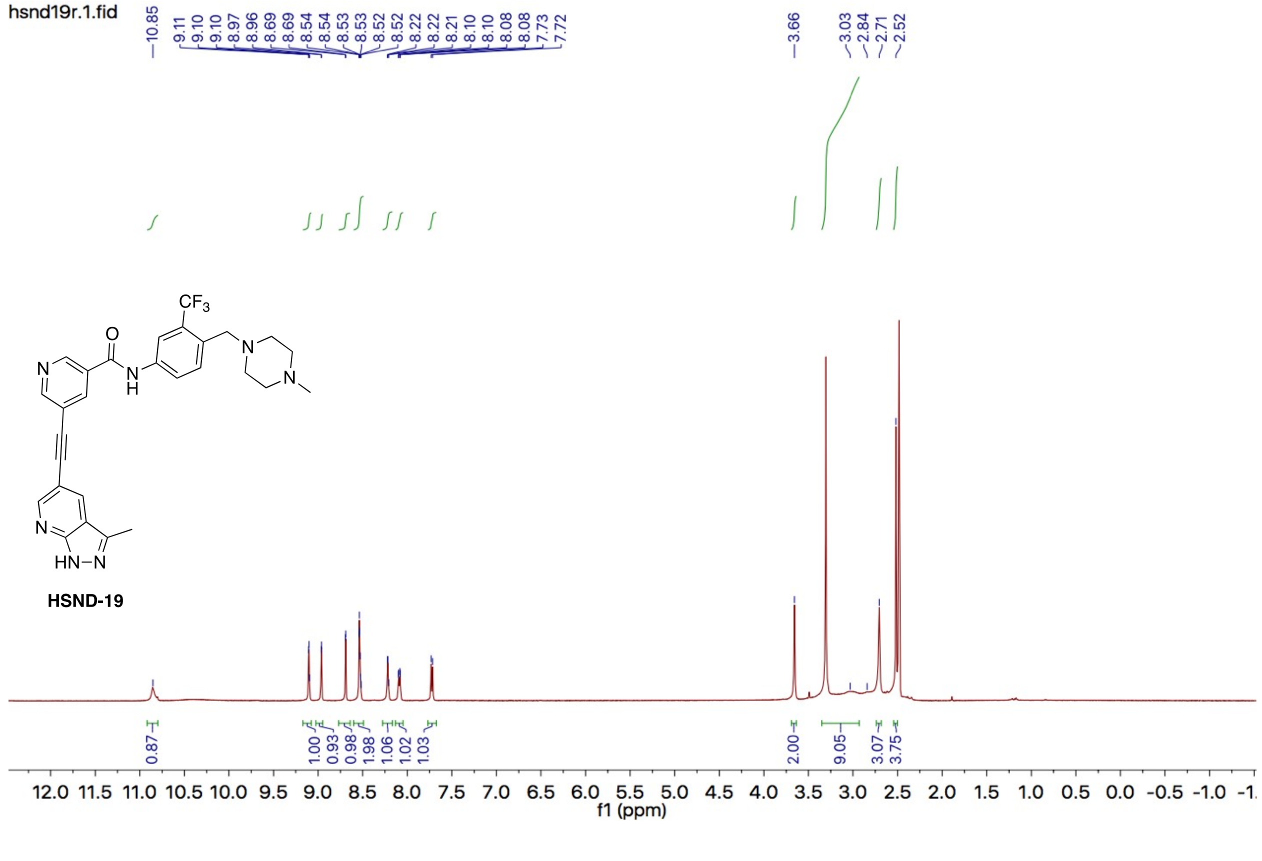

HSND-19

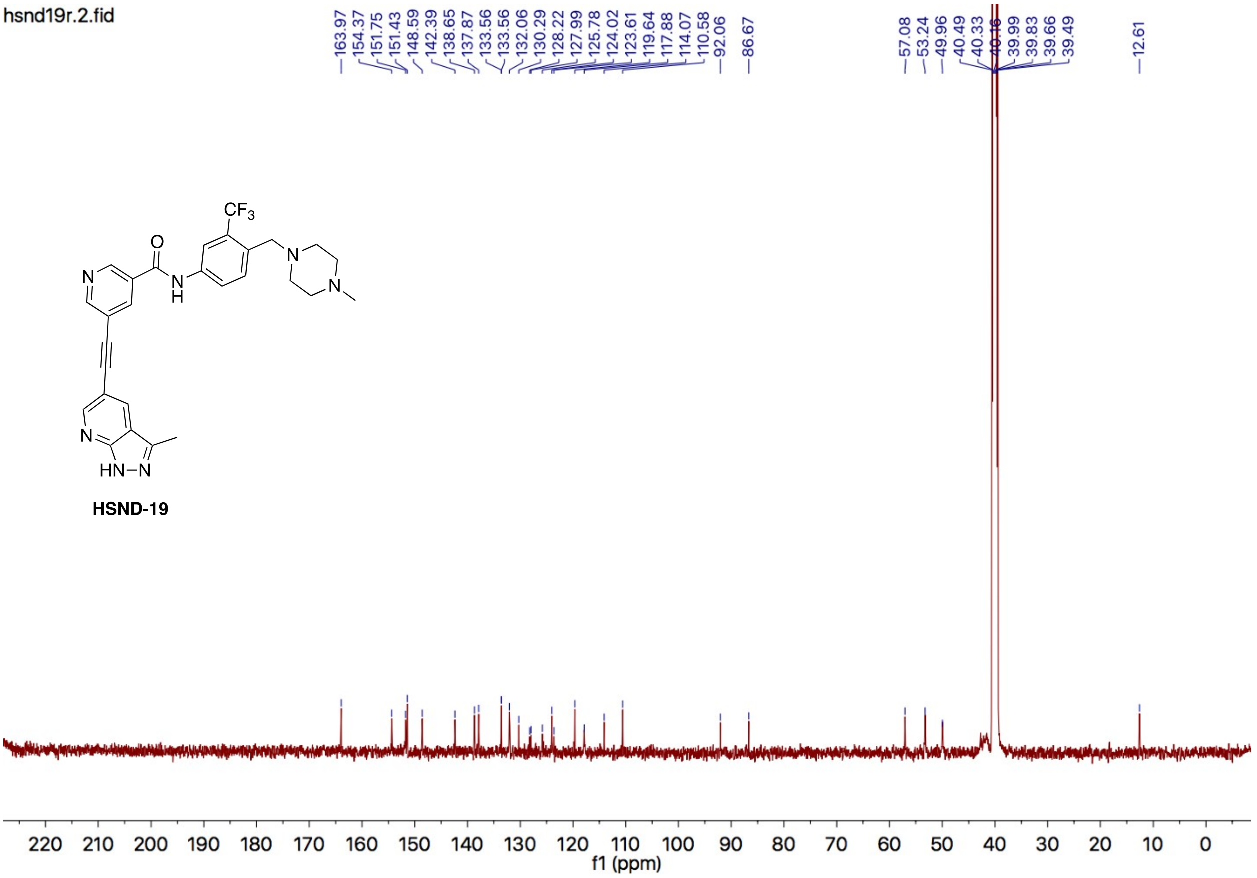

CF3
O
N
NH
N
N
N
HN–N
HSND-19
hsnd19r.2.fid
163.97
154.37
151.75
151.43
148.59
142.39
138.65
137.87
133.56
133.56
132.06
130.29
128.22
127.99
125.78
124.02
123.61
119.64
117.88
114.07
110.58
92.06
86.67
57.08
53.24
49.96
40.49
40.33
40.16
39.99
39.83
39.66
39.49
12.61
220 210 200 190 180 170 160 150 140 130 120 110 100 90 80 70 60 50 40 30 20 10 0
f1 (ppm)
